# The effects of thymidylate synthase 3'UTR genotype on methylation of tumor-specific genes promoter in 22 colorectal cancer patients from southern Iran

**DOI:** 10.22099/mbrc.2023.48009.1850

**Published:** 2024

**Authors:** Maryam Niknam, Fakhraddin Naghibalhossaini, Mozhdeh Zamani, Seyed Vahid Hosseini, Pooneh Mokarram

**Affiliations:** 1 Department of Biochemistry, Shiraz University of Medical Sciences, School of Medicine, Shiraz, Iran; 2 Autoimmune Diseases Research Center, Shiraz University of Medical Sciences, School of Medicine, Shiraz, Iran; 3 Autophagy Research Center, Shiraz University of Medical Sciences, Shiraz, Iran; 4 Colorectal Research Center, Shiraz University of Medical Sciences, Shiraz, Iran; 5 Autophagy Research Center, Department of Biochemistry, School of Medicine, Shiraz University of Medical Sciences, Shiraz, Iran

**Keywords:** Thymidylate synthase, Methylation, DNA methyltransferase, Histone deacetylase, Colorectal cancer

## Abstract

To investigate the effects of *thymidylate synthase (TS) *3'UTR genotype on promotor methylation of tumor-related genes in 22 patients with sporadic colorectal cancer (CRC) from southern Iran. We evaluated the correlations of *TS *3'UTR genotype with promoter methylation of *hTERT, hMLH1, MSH2, MMP2, CDH1, p14, p16,* and *p21* genes in CRC patients. The polymorphism of *TS* 3′UTR was evaluated through mutagenically specific PCR. The genes promoter methylation was determined using methylation-specific PCR. For 10 patients, the gene expression profile of epigenetic regulating enzymes, *histone deacetylases (HDACs)* and *DNA methyltransferases* *(DNMTs),* was also examined in both tumor and normal adjacent tissues by quantitative real time PCR. There was a significant association between the *hMLH1* methylation and age of patients (*P*= 0.039) and also between *MSH2* methylation and tumor site (*P*= 0.036). There was insignificant association between gene-specific methylation and *TS* 3′UTR genotype. However, all polymorphic genotypes of *TS* were associated with higher methylation of *hMLH1* and *CDH1* and lower methylation of* MSH2*. The -6bp/+6bp (heterozygous mutant) and [-6bp/+6bp, +6bp/+6bp] (homozygous mutant) genotypes resulted in higher methylation of *p16*, and -6bp/+6bp and [-6bp/+6bp, +6bp/+6bp] genotypes were correlated with lower methylation of *MMP2*. The overexpression of epigenetic enzymes, *HDACs* and *DNMTs, *was also demonstrated. There was no association between DNMTs transcript levels and gene-specific hypermethylation. The polymorphic TS genotypes, especially -6bp/+6bp, could affect methylation frequencies of studied genes. Moreover, promoter methylation status was not dependent on *DNMTs* gene expression. Large sample size studies may contribute to validate these findings.

## INTRODUCTION

As a critical health burden, colorectal cancer (CRC) is a leading cause of mortality and morbidity worldwide [1]. CRC is among the most frequent cancers in Iranian population [2]. It has been reported that in Iran CRC is considered as the third most frequent cancer in men and the fourth one in women [3]. It is a multistep process that comes from the accumulation of numerous genetic and epigenetic aberrations under contextual effect. Genetic and epigenetic characteristics are highly prominent in CRC, and there is a need to be evaluated concurrently in order to identify predictive biomarkers for chemotherapy [4]. 

Recently, the impacts of nutrients on gene expression by cooperation with genetic polymorphisms and modulation of DNA methylation have been taken into consideration. It has been suggested that the homeostasis disruption of the vitamin dependent one-carbon metabolism may influence the cancer risk [5]. Studies on colorectal cancer have indicated that polymorphisms of key genes contributing to folate metabolism are likely to be correlated with the CRC risk, possibly through their effects on DNA methylation or synthesis [6]. 

As a crucial enzyme involved in folate metabolism, thymidylate synthase (TS) catalyzes the conversion of deoxyuridine monophosphate (dUMP) to deoxythymidine monophosphate (dTMP), as a rate-limiting reaction in the synthesis of thymidine. The *TS* gene contains two functionally relevant polymorphisms. In the 5′ untranslated region (5′UTR) enhancer region(TSER), lower frequencies of a 28 bp sequence lead to decreased *TS* expression [7, 8]. Moreover, the del6 polymorphism of the *TS *3'UTR (3′UTR 1494delTTAAAG) results in reduced *TS* mRNA stability and its intratumoral expression [9, 10]. Several studies have investigated the correlation of the *TS* 3′UTR polymorphism and CRC risk, but the results are controversial [10, 11]. 

Histone modifications, DNA methylation, and non-coding RNAs are the epigenetic modifications with crucial impacts on tumor development from initiation to metastasis [1]. It has been found that DNA methylation and histone deacetylation can act synergistically in the epigenetic regulation of cancer-associated genes [12]. Histone deacetylases (HDACs), in collaboration with DNA methyltransferases (DNMTs), may have an important impression in silencing of tumor suppressor genes (TSGs). Such effect was found for the epigenetic regulation of *MLH1* in CRC cells [13, 14]. A large body of evidence demonstrated that *DNMTs* and *HDACs* were overexpressed in CRC [15-20]. Histone modifications have been found to regulate the gene expression and mediate CRC carcinogenesis, in cooperation with DNA methylation [14]. 

Although numerous studies have described the correlation of the *TS* 3′UTR polymorphism and CRC risk, they have gained discrepant results and the function of this *TS* variant has not been completely understood [10, 11, 21]. It has been reported that variation in TS functions might contribute to carcinogenesis through deviant DNA methylation [22]. In the present study, we evaluated the association between the 3'UTR genotype of* TS *and methylation status of 8 tumor-related genes, including h*TERT, *h*MLH1, MSH2, MMP2, CDH1, p14, p16,* and *p21 *in CRC patients to define the possible molecular mechanisms that associate the *TS* 3′UTR genotype and CRC susceptibility. 

The evaluation of the expression patterns and associations of DNMTs and HDACs is critical to improve the clinical cancer treatment. The enhanced knowledge of epigenetic control of gene transcription in CRC pathogenesis has led to identification of epigenetic diagnostic and therapeutic biomarkers for CRC [14]. In our study, we analyzed the levels of *DNMTs* expression (*DNMT1*,* DNMT3a*, and *DNMT3b*) and *HDACs* (*HDACs1-4* and *SIRT1*) in sporadic CRC patients. There are great controversies regarding to the effect of enhanced *DNMTs* expression on deviant DNA methylation and CIMP phenotype of colon cancer [23-25]. In this study, we also investigated whether there were associations between transcript levels of three DNMT enzymes *(DNMT1*, *DNMT3a* and *DNMT3b*) and gene-specific promoter methylation in CRC patients. 

## MATERIALS AND METHODS

**Patients and tumor specimens: **We collected the tumor and adjacent normal tissues that were surgically resected from 22 CRC patients of one university hospital in Shiraz, southern of Iran, from 2021 to 2022. The informed consent was obtained from each subject or subject’s guardian. This study was ethically approved by the institutional ethics committee (Ethical approval ID: IR.SUMS.REC.1402.185). Immediately after surgical resection, we snap frizzed and stored the tumor and normal tissues at -80ºC. An expert pathologist performed the histological diagnosis and determined the proper tissue sections for DNA and RNA extraction and subsequent molecular studies. We obtained the patients’ clinicopathological characteristics from hospital records.

**DNA extraction and TS 3′UTR genotyping:** The standard phenol-chloroform extraction method was performed for genomic DNA extraction from tumor and normal specimens. The polymorphism of the *TS* 3′UTR at bp 1494 was verified through restriction fragment length polymorphism (RFLP) technique. A DraI restriction site was created as a result of the presence of the 6 bp. The amplification of polymorphic fragment was performed through PCR by the use of primers 5′CAAATCTGAGGGAGCTGAGT3′ and 5′CAGATAAGTGGCAGTACAGA3′ in a 50 µl reaction volume of 100 ng of genomic DNA, 300 nM of each primer, 1x PCR buffer, 150 µM deoxynucleotide triphosphates (dNTPs), and 2.5 mM MgCl2 and 1 unit DNA polymerase (SinaClon, Iran). The PCR cycling included a precycling heat activation at 94°C for 5 minutes, followed by 30 cycles of 94°C for 30 sec, 58°C for 45 sec, 72°C for 45 sec, and final extension cycle of 72°C for 5 minutes. Thereafter, through restriction enzyme (*Dra*I), the amplified products were digested and then separated on a 3% agarose gel electrophoresis. The wild-type allele had 70 and 88 bp PCR products and the product of mutant allele was 148 bp [10]. 

**Methylation-specific PCR (MSP) analysis of the gene promoter methylation:** The promoter methylation status of 8 candidate tumor-associated genes (h*TERT*, h*MLH1*, *MSH2,* *MMP2*, *CDH1,* *p14*, *p16*, and *p21*) in normal and tumor tissues was determined by MSP method [26]. Briefly, 1 µg of the genomic DNA was treated with sodium bisulfite, and then PCR amplification was done using two primer sets (Table 1) specific for both methylated and unmethylated CpG islands in the genes promoter. The MSP products were detected using 1.5 % agarose gel electrophoresis with UV illumination. 

**Table 1:** Sequence and annealing temperature of the primers used for methylation-specific PCR

**Gene**	**Forward primer**	**Reverse primer**	**AnnealingT (°C)**	**Productsize (bp)**
*hTERT*	U: 5′-AGTTTTGGTTTTGGTTATTTTTGT-3′ M: 5′-AGTTTTGGTTTCGGTTATTTTCGC-3′	5′-AACGTAACCAACGACAACACCT-3′ 5′-AACGTAACCAACGACAACACC-3′	58	132 122
*hMLH1*	U: 5′-TTTTGATGTAGATGTTTTATTAGGGTTGT-3′ M: 5′-ACGTAGACGTTTTATTAGGGTCGC-3′	5′-ACCACCTCATCATAACTACCCACA-3′ 5′-CCTCATCGTAACTACCCGCG-3′	58	118 124
*MSH2*	U: 5′-GGTTGTTGTGGTTGGATGTTGTTT-3′ M: 5′-TCGTGGTCGGACGTCGTTC-3′	5′-CAACTACAACATCTCCTTCAACTACACCA-3′ 5′-CAACGTCTCCTTCGACTACACCGG-3′	58	144 133
*MMP2*	U: 5′-GTGGTTATATGTATTGAGTTAGTGATTTTTGGGTG-3′ M: 5′-TATCGAGTTAGCGATTTTCGGGC-3′	5′AAAAAACAAAACACCCTCAAAAAACCCATAACA-3′ 5′-CGCCCTCAAAAAACCCGTAAACG-3′	53	96 96
*CDH1*	U: 5′-TAATTTTAGGTTAGAGGGTTATTG-3′ M: 5′-TTAGGTTAGAGGGTTATCGCG-3′	5′-CACAACCAATCAACAACAC-3′ 5′-TAACTAAAAATTCACCTACCGA-3′	53 57	97 116
*p14ARF*	U: 5′-TTTTTGGTGTTAAAGGGTGGTGTAGT-3′ M: 5′-GTGTTAAAGGGCGGCGTAGC-3′	5′ CACAAAAACCCTCACTCACAACAA-3′ 5′-AAAACCCTCACTCGCGACGA-3′	60	155 145
*p16INK4a*	U: 5′-TTATTAGAGGGTGGGGTGGATTGT-3′ M: 5′- TTATTAGAGGGTGGGGCGGATCGC-3′	5′-CAACCCCAAACCACAACCATAA-3′ 5′-GACCCCGAACCGCGACCGTAA-3′	60	151 149
*p21*	U: 5′-TTTTTGTAGTATGTGAGGTTTTGG-3′ M: 5′-TGTAGTACGCGAGGTTTCG-3′	5′-AACACAACTCAACACAACCCTA-3′ 5′-TCAACTAACGCAACTCAACG-3′	54	200 202

**RNA extraction and quantitative Real Time-PCR (qRT-PCR):** Total RNA was extracted from normal and tumor CRC tissues using a BIOZOL RNA isolation kit (Bioflux-Bioer, China), according to the manufacturer's protocols. The integrity of the extracted RNAs was confirmed by electrophoresis on denaturing agarose gels (1.5%) with 2% formaldehyde. 

The relative transcript levels of the target genes (*HDAC1*,* 2, 3, 4, SIRT1, DNMT1, DNMT3a,* and *DNMT3b*) in normal and tumor tissues were evaluated using real-time RT-PCR assay as described previously [27]. Briefly, the complementary DNA (cDNA) was synthesized by reverse transcription (RT) of 2 microgram total RNA by the use of the M-MuLV reverse transcriptase and oligodT primers in a 20 μl reaction volume according to the instructions provided by the manufacturer (Cinagene, Iran). Real time PCR quantification of each gene was done on 1 µl cDNA in a 25µl reaction mixture using gene-specific primer sets (Table 2) and SYBR Green master mix (Ampliqon, Danmark) in a QuantStudio™ 3 Real Time PCR System (Applied Biosystems, USA). The amplification of genes was performed in triplicate with a precycling heat activation at 95°C for 10 min, followed by 40 cycles of 95°C for 15 s, 60°C for 30 s, 72°C for 30 s, and a final extension at 72°C for 10 min. The β-Actin, as an internal control gene, was used to normalize the expression level of target genes using the 2^-ΔΔCT^ formula. We also aimed to associate the expression profiles of three DNMTs with gene-specific DNA methylation changes in CRC tissues.

**Table 2:** Primer sequences used for real time RT-PCR

**Gene**	**Forward primer**	**Reverse primer**	**Product size (bp)**
*β-Actin*	5΄-AATCGTGCGTGACATTAAG-3΄	5΄-GAAGGAAGGCTGGAAGAG-3΄	178
*HDAC1*	5′-GGAAATCTATCGCCCTCACA-3′	5′-AACAGGCCATCGAATACTGG-3′	168
*HDAC2*	5′-TAAATCCAAGGACAACAGTGG-3′	5′-GGTGAGACTGTCAAATTCAGG-3′	89
*HDAC3*	5′-TAGACAAGGACTGAGATTGCC-3′	5′-GTGTTAGGGAGCCAGAGCC-3′	120
*HDAC4*	5′-GGTTTATTCTGATTGAGAACTGG-3′	5′-ATTGTAAACCACAGTGCTCGC-3′	146
*SIRT1*	5′-TGCGGGAATCCAAAGGATAATTCAGTGTC-3′	5′-CTTCATCTTTGTCATACTTCATGGCTCTATG-3′	200
*DNMT1*	5′-CGACCACTTTGTCAAGCTCA-3′	5′-AGGGGTCTACATGGCAACTG-3′	103
*DNMT3a*	5′-TATTGATGAGCGCACAAGAGAGC-3′	5′-GGGTGTTCCAGGGTAACATTGAG-3′	111

**Statistical analyses: **The SPSS version 18 (SPSS Inc., Chicago, IL) was used to perform the statistical analyses. Data are reported as mean±standard deviation (SD). The difference between the two groups was analyzed by an unpaired student’s t-test. The *P*-value below 0.05 (*P*<0.05) was considered statistically significant. The Chi square and Fisher’s exact test was performed to evaluate the associations between loci methylation and genotypic and clinicopathologicl characteristics of patients.

## RESULTS

The clinicopathological features of the study participants are shown in Table 3. Twenty two patients was enrolled in the study. Patients were more likely to be males (68.2%) and older than 60 years (63.6%). About 86.4% (19) of the patients had distal CRC and 13.6 percent (13.6%) (3) had proximal CRC. With respect to tumor stage, 4 tumors (18.2%) were in stage I, 10 patients (45.5%) had tumors with stage II, and 8 tumors (36.4%) were in stage III. About tumor differentiation, fourteen patients (63.6%) had well-differentiated tumors, while 6 (27.3%) and 2 (9.1%) of them were moderate and poorly differentiated, respectively.

**Figure 1:**
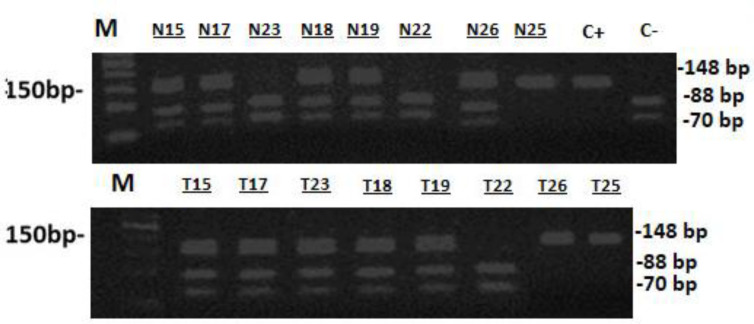
Representative examples of MS-PCR for genotyping of 3′UTR of the TS gene.

The presence of the SNP at codon 1494 of the *TS* 3′UTR was analyzed in CRC cases. The *TS* genotyping was evaluated by PCR-RFLP. Figure 1 illustrates examples of the *TS* genotyping. For 10 patients, we performed genotyping in both cancer and the adjacent normal tissues, in 7 (70%) cases’ the results were similar in both samples.

Genotype frequencies and patients′ characteristics in relation to the *TS* 3′UTR genotypes are summarized in Table 4. Of 22 cases, 3 (13.6%) had the -6bp/-6bp (wildtype), 14 (63.6%) the -6bp/+6bp (heterozygous mutant), and 5 (22.7%) the +6bp/+6bp (homozygous mutant) genotype; allele frequencies were mutant: 86.4 % and wild type: 13.6%. The distribution of the *TS *3′UTR genotypes among both normals (-6bp/-6bp, 10%; -6bp/+6bp, 60%; +6bp/+6bp, 30%) and tumors (-6bp/-6bp, 13.6%; -6bp/+6bp, 63.6%; +6bp/+6bp, 22.7%) agreed with that expected from the Hardy–Weinberg equilibrium (χ^2^=0.11, *P*=0.945; χ^2^=1.02, *P*=0.599, respectively).

**Table 3:** Distributions of selected characteristics of CRC patients

**Variables**	**Total=22 n** **(%)**
Age:	
< 60 years ≥ 60 years	8 (36.4) 14 (63.6)
Sex:	
Male Female	15 (68.2) 7 (31.8)
Stage:	
I II III	4 (18.2) 10 (45.5) 8 (36.4)
Site:	
Distal Proximal	19 (86.4) 3 (13.6)
Differentiation:	
Well Moderate Poor	14 (63.6) 6 (27.3) 2 (9.1)

Using restriction enzyme (DraI), the amplified products were digested and then separated on a 3% agarose gel electrophoresis. The wild-type allele had 70 and 88 bp PCR products, and the product of mutant allele was 152 bp. For 10 patients, we performed genotyping in both the tumor (T) and adjacent normal (N) tissues M: DNA size marker. C+: Control positive and C-: Control negative.

As presented in Table 4, there was no significant association between *TS* 3′UTR genotypes and clinic-pathological characteristics of the patients (*P* >0.05). 

**Figure 2:**
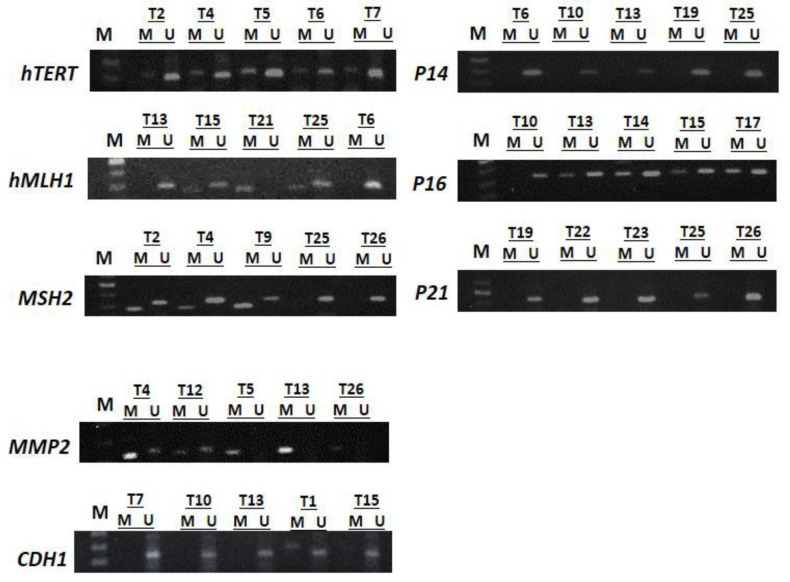
MSP results of promoter methylation status of *hTERT,* *hMLH1*, *MSH2, p14, p16, p21, MMP2 *and* CDH1 *genes in CRCs. U: unmethylated genes; M: methylated genes. Lane 1 shows the 50 bp DNA marker

As described in the previous section, CpG islands hypermethylation in tumors was analyzed by MSP. Representative examples are demonstrated in Figure 2. In Table 5, the correlations of genes promoter methylation, *TS *3′UTR genotype, and other clinico-pathological features of CRC cases are summarized.

The most frequent methylated locus was *hTERT *(100%; 22 of 22), followed by *MMP2* (90.9%; 20 of 22), *p16* and *hMLH1* (77.3%, 17 of 22), *CDH1* (45.5%, 10 of 22), and *MSH2* (36.4%; 8 of 22). None of the studied patients had methylation in the *p14* and *p21* genes. We observed no simultaneous ptomoter hypermethylation of all eight studied genes in these patients. Because of the equal methylation status in *hTER*, *p14* and *p21*, we could not enter these genes to subsequent analyses. Nine out of 22 (40.9%) tumors were methylated in 5 genes, and 11 out of 22 (50%) had methylation in 4 genes (Table 6).

**Table 4:** Patients’ characteristics according to TS 3′UTR polymorphis

**Variables**	**Number**	**-6bp/-6bp**	**-6bp/+6bp**	**+6bp/+6bp**	*P
Cases, n (%) Total	22	3 (13.6)	14 (63.6)	5 (22.7)	
					
Age <60 ≥60	8 14	1 (12.5) 2 (14.3)	6 (75) 8 (57.1)	1 (12.5) 4 (28.6)	0.812
					
Sex Male Female	15 7	1 (6.7) 2 (28.6)	11 (73.3) 3 (42.9)	3 (20) 2 (28.6)	0.354
					
Site Proximal Distal	3 19	0 (0) 3 (15.8)	2 (66.7) 12 (63.2)	1 (33.3) 4 (21.1)	1
					
Tumor Stage I II III	4 10 8	0 (0) 1 (10) 2 (25)	2 (50) 7 (70) 5 (62.5)	2 (50) 2 (20) 1 (12.5)	0.636
					
Differentiation Well Moderate Poor	14 6 2	2 (14.3) 1 (16.7) 0 (0)	8 (57.1) 4 (66.7) 2 (100)	4 (28.6) 1 (16.7) 0 (0)	1

*P: *P* value from Fisher′s exact test.

As described in Table 5, there was a significant association between the *hMLH1* methylation and age of patients (*P*=0.039); the *hMLH1* methylation was significantly greater in patients aged≥60 years old as compared to<60 year old patients. Moreover, a significant correlation was also found between *MSH2* methylation and the tumor site (*P*=0.036), as, that the methylation was significantly higher in distal tumors than proximal ones.

**Table 5:** Associations between genes promoter methylation and clinico-pathological features of CRC patients

**Variables**	**h** ***MLH1, n*** M U * ***P***			***MSH2, n*** M U * ***P***				***MMP2, n*** M U * ***P***				***CDH1, n*** M U * ***P***				***p16, n*** M U * ***P***			
Total	17 5			8 14				20 2				10 12				17 5			
Age <60 (8) ≥60 (14)	4 13	4 1	**0.039**		5 3	3 11	0.081		7 13	1 1	1		4 6	4 8	1		8 9	0 5	1.115	
															
Sex Male (15) Female (7)	10 7	5 0	0.135		6 2	9 5	1		14 6	1 1	1		9 1	6 6	0.074		12 5	3 2	1	
															
Site Proximal (3) Distal (19)	1 16	2 3	0.117		3 5	0 14	**0.036**		2 18	1 1	0.260		3 7	0 12	0.078		2 15	1 4	1	
															
Tumor Stage I (4) II (10) III (8)	2 8 7	2 2 1	0.354		2 4 2	2 6 6	0.727		3 9 8	1 1 0	0.459		3 4 3	1 6 5	0.624		3 8 6	1 2 2	1	
															
Differentiation Well (14) Moderate (6) Poor (2)	10 5 2	4 1 0	1		6 2 0	8 4 2	0.812		12 6 2	2 0 0	1		9 1 0	5 5 2	0.074		10 5 2	4 1 0	1	
															
TYMS genotype -6bp/-6bp (3) -6bp/+6bp (14) +6bp/+6bp (5)	2 10 5	1 4 0	0.464		2 5 1	1 9 4	0.671		3 14 3	0 0 2	0.056		1 7 2	2 7 3	1		2 12 3	1 2 2	0.350	
-6bp/-6bp (3) -6bp/+6bp+ +6bp/+6bp (19)	2 15	1 4	1		2 6	1 13	0.527		3 17	0 2	1		1 9	2 10	1		2 15	1 4	1	

**Table 6:** Methylated gene profiles in patients with methylation in 4 and 5 genes

**4 Methylated Genes**	
N (%)	Gene Profile
7 (63.6)	h*TERT*, h*MLH1*, *MMP2*, *p16*
2 (18.2)	h*TERT*, *MSH2*, *MMP2*, *CDH1*
2 (18.2)	h*TERT*, h*MLH1*, *MMP2*, *CDH1*
**5 Methylated Genes**	
N (%)	Gene Profile
3 (33.3)	h*TERT*, *MSH2*, *MMP2*, *CDH1*, *p16*
3 (33.3)	h*TERT*, h*MLH1*, *MMP2*, *CDH1*, *p16*
2 (22.2)	h*TERT*, h*MLH1*, *MSH2*, *MMP2*, *p16*
1 (11.1)	h*TERT*, h*MLH1*, *MSH2*, *CDH1*, *p16*

Table 7 summarizes the distribution of gene-specific methylation (%) in the *TS* genotypes. It was demonstrated that in comparison with the -6bp/-6bp genotype (66.7%) with wild type allele, the other 3 genotypes, containing mutant allele, had higher *hMLH1* methylation frequencies (71.4% in the -6bp/+6bp, 100% in the +6bp/+6bp, and 78.9% in the -6bp/+6bp + +6bp/+6bp genotype). Unlike *hMLH1*, our results revealed that the *MSH2* methylation frequencies were lower in patients with mutant allele of *TS* (35.7% in the -6bp/+6bp, 20% in the +6bp/+6bp, and 31.6% in the -6bp/+6bp + +6bp/+6bp genotype) as compared to patient who had wild type allele (66.7%). As to *MMP2* gene, all subjects with the -6bp/-6bp and -6bp/+6bp genotypes were methylated for this gene, while 60% of patients with the +6bp/+6bp and 89.5% of patients with the -6bp/+6bp + +6bp/+6bp genotype had *MMP2* methylation. For *CDH1* gene, there were higher methylation frequencies in patients with the -6bp/+6bp (50%), +6bp/+6bp (40%), and -6bp/+6bp + +6bp/+6bp (47.4%) genotypes than in the -6bp/-6bp genotype (33.3%). Although patients with the +6bp/+6bp genotype had a slightly lower *p16* methylation (60%), in subjects with the -6bp/+6bp (85.7%) and -6bp/+6bp + +6bp/+6bp (78.9%) genotypes, higher *p16* methylation was found compared to the -6bp/-6bp genotype (66.7%). We also investigated if the percentage of cases with the same methylation frequencies (methylated in 4 or 5 genes) was different between the *TS* genotypes. We found that the frequency of patients with 4 methylated genes was lower in the -6bp/+6bp (51.7%), +6bp/+6bp (20%), and -6bp/+6bp + +6bp/+6bp (47.4%) than in the -6bp/-6bp genotype (66.7%). However, the percentage of cases with 5 methylated genes was higher in the -6bp/+6bp (42.9%), +6bp/+6bp (40 %), and -6bp/+6bp + +6bp/+6bp (42.1%) as compared to the -6bp/-6bp genotype (33.3%).

**Table 7:** Distributions of gene-specific methylation between different *TS* 3′UTR genotypes

**Methylation Positive**							
**Variables**	**h** ***MLH1***	***MSH2***	***MMP2***	***CDH1 ***	***p16***	**4** ^a^ ** Methylated genes**	**5** ^b^ ** Methylated genes**
**-6bp/-6bp (3)**	2 (66.7)	2 (66.7)	3 (100)	1 (33.3)	2 (66.7)	2 (66.7)	1 (33.3)
**-6bp/+6bp (14)**	10 (71.4)	5 (35.7)	14 (100)	7 (50)	12 (85.7)	8 (51.7)	6 (42.9)
**+6bp/+6bp (5)**	5 (100)	1 (20)	3 (60)	2 (40)	3 (60)	1 (20)	2 (40)
**[-6bp/+6bp, +6bp/+6bp] (19)**	15 (78.9)	6 (31.6)	17 (89.5)	9 (47.4)	15 (78.9)	9 (47.4)	8 (42.1)

a: Eleven patients with a panel of 4 methylated genes as described in Table 6.

b: Nine patients with a panel of 5 methylated genes as described in Table 6.

**Figure 3:**
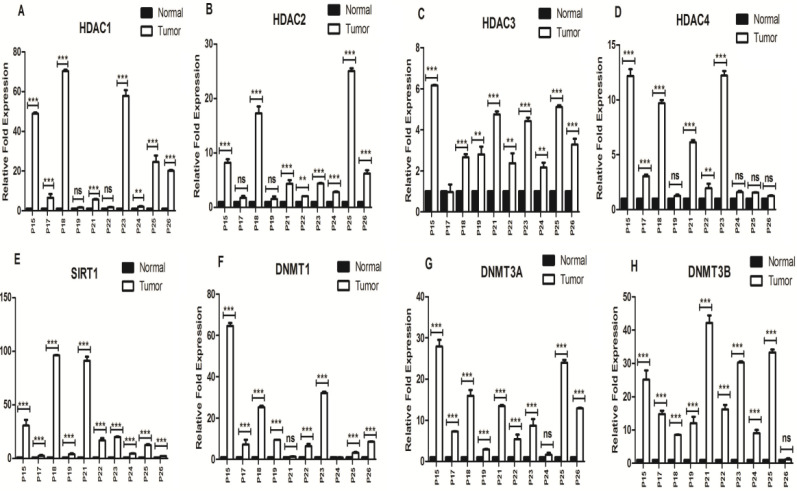
qRT-PCR analysis of relative expressions of HDAC1, 2, 3, 4 (A-D), SIRT1 (E), and DNMT1 (F), 3a (G) and 3b (H) in the CRC tissue specimens (n=10), compared with their respective normal samples.

The expressions of the *HDACs* (*HDAC 1, 2, 3, 4* and *SIRT1*), *DNMT1, DNMT3a,* and *DNMT3b* transcripts were analyzed using qRT-PCR and shown in Figure 3A-H. The relative expressions of *HDAC 1, 2, 3, 4* and *SIRT1* mRNA were significantly higher in CRC tissues as compared with normal ones, in particular for *SIRT1* (*P*<0.001). There were significantly higher *DNMT1, DNMT3a,* and *DNMT3b* expressions in CRCs than in normal tissues, especially for *DNMT3b* (*P*<0.001; except for P26). 

In a few number of cases, the gene upregulation was not statistically significant (including P19 for *HDAC1*; P17 and P19 for *HDAC2*, P19, P24, P25 and P26 for *HDAC4*) and in P17 there were no differences in the transcript level of *HDAC3* between the tumor and normal colorectal tissues. With respect to *SIRT1*, significant overexpression of this gene was found in all tumor tissues as compared with normal ones.

With regard to *DNMTs*, it was found that in some cases, the gene overexpression was not statistically significant (including P21 for *DNMT1*; P24 for *DNMT3a*; and P26 for *DNMT3b*) and in P24, no differences were detected in the *DNMT1* expression between tumor and normal tissues.

**Figure 4:**
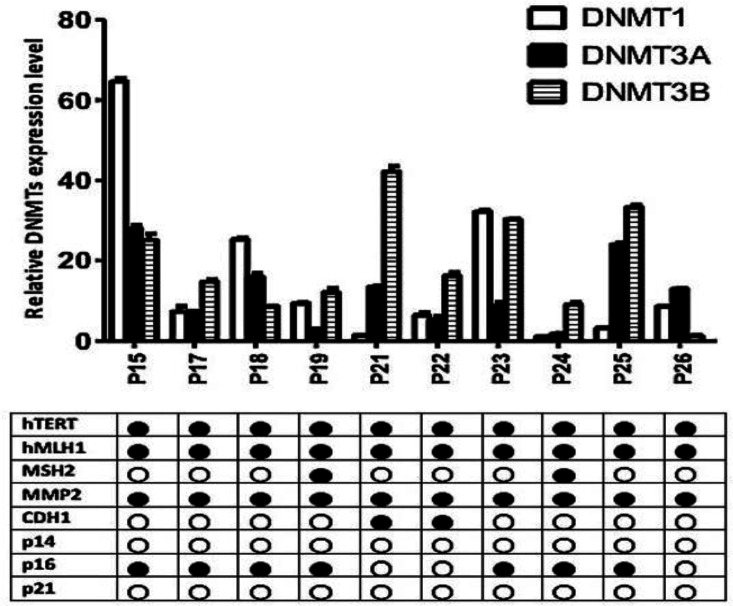
Association of expression levels of *DNMT1, DNMT3a* and *DNMT3b* with promoter methylation of 8 genes in ten CRC patients.

As shown in Figure 4, there was no association between methylation of 8 CpG islands evaluated in the CRC tissues and transcript levels of the three *DNMTs*. P15 and P24 had the highest and lowest *DNMTs* transcript levels, respectively. Three out of 8 genes (h*TERT, *h*MLH1,* and *MMP2*) were found to be hypermethylated in all studied patients. The number of methylated promoters was higher in P19 and P24 (five out of 8 studied genes), with the moderate (8.133) and the lowest (3.875) levels of *DNMTs* expressions, respectively. Moreover, the lowest number of methylated genes was detected in P26, with moderate levels of *DNMTs* expression (7.562). Differential analysis of CpG islands indicated that only P19 and P24, with moderate and low transcripts levels of *DNMTs*, had *MSH2* promoter methylation. However, the promoter methylation of the *p16* gene was detected in low to high DNMTs expressors.

The internal control gene, β-actin, was used to normalize the gene expression levels. The data are presented as means ± SD of two independent assays in triplicate. *p < 0·05, **p < 0.01, and ***p<0.001, as described by Unpaired T-Test.

Methylation status of 8 genes in each patient is demonstrated in the chart below. ●: methylated; ○: unmethylated

## DISCUSSION

As a leading cause of mortality and morbidity worldwide, CRC is a multistep disease that arises from the accumulation of genetic and epigenetic abnormalities under microenvironmental effect [1]. Currently, the nutrient influences on gene expression by cooperation with genetic polymorphisms and modulation of DNA methylation has received a great interest [28]. Although the complex interplays among ‘‘one-carbon metabolism, genetic polymorphisms, and the promoter methylation status of the selected genes in CRC have been verified by previous studies , there is a need to further clarify this concern in additional researches [29]. In the current study, we analyzed the association between the *TS *3'UTR genotype and promoter methylation status of 8 tumor-specific genes, *hTERT, hMLH1, MSH2, MMP2, CDH1, p14, p16,* and *p21* in 22 unselected series of sporadic CRC patients. 

The major genotype population in the cases was the heterozygous -6bp/+6bp genotype (Table 4). As to allele frequencies, 86.4% of the patients were mutant and 13.6% of them had wild type allele. TS is the key target of 5-FU, a chemotherapeutic drug used in all standard adjuvant chemotherapies for CRC. Initial evidence supported the pharmacogenetic impacts of *TS* polymorphisms on both drug efficacy and toxicity of 5-FU [30, 31]. Since *TS* was polymorphic in 86.4% of patients, it could be concluded that the observed metastasis (P7, P9, and P22) and even death resulting from the disease (P4, P17, P21, P23, and P26) in some cases might have resulted from the presence of mutated allele of *TS* and subsequently drug resistance.

In all patients, there was no association between the existing *TS* genotype and their clinico-pathological characteristics (Table 4). The most frequent methylated locus was *hTERT *(100%; 22 of 22). It has been evidenced that in most cancer cell lines and tissues, there is dense hypermethylation in the *hTERT* promoter [32, 33]. Interestingly, unlike the usual effect of DNA methylation on gene expression, *hTERT* promoter methylation is directly associated with gene expression [34]. It could be explained by the lack of methylation near the transcription start site of *hTERT* [32].

Moreover, in the same line with previous studies [35, 36], *p14* and *p21 *genes were unmethylated in all patients. It has been demonstrated that in sporadic CRC, the dense hypermethylation of *p14* could not be considered as a common phenomenon [35]. It is also evident that the *p21* promoter had no tumor-associated DNA methylation. The possible causes for these findings were the fact that the transcript levels of these genes did not alter in CRC, or the changes in gene expression did not mediate by DNA methylation [36].

Interestingly, 11 out of 22 (50%) patients had 4 methylated genes (among them, 63.6% were methylated in h*TERT, *h*MLH1, MMP2,* and* P16* genes). Although the patients with this methylated panel were at stages II and III, a non-significant association was observed between the occurrence of this methylated gene and CRC stage that may be the result of the small sample size. Therefore, this panel of methylation markers could be suggested as a diagnostic marker for stage II and III CRC to be evaluated in future studies. 

In our study, the frequency of tumors with *hMLH1* gene methylation was higher in patients aged ≥ 60 years old (*P*= 0.039), and a significant excess of *MSH2* methylation was also found in distal tumors (*P*= 0.036) (Table 5). There was no association between methylation status of other genes and the clinico-pathological characteristics under study. 

We confronted the *TS* genotype with the gene-specific methylation of tumors using the -6bp/-6bp genotype as the control group (Table 7). Our results demonstrated that the polymorphic genotypes of *TS*, especially the -6bp/+6bp genotype, were associated with higher methylation frequencies of h*MLH1*, *CDH1*, and *p16* genes, as well as lower methylation frequencies of *MSH2* and *MMP2* genes. In sporadic CRC, the promoter hypermethylation of *hMLH1* and *MSH2* are considered as a hallmark of MSI [37]. As a tumor suppressor gene, *p16* is a CDK inhibitor with critical role in cell cycle regulation [38]. Moreover, h*MLH1 *and *p16* methylation were included in the panel of markers used to assess the CIMP phenotype [39]. *CDH1 *and *MMP2* genes are metastasis prediction markers [40]. These findings present the *TS* 3′UTR polymorphism as a CRC risk factor that contributes to CRC carcinogenesis by epigenetic (promoter hypermethylation) regulation of MSI, CIMP, metastasis, and tumor cell cycle. It is noteworthy that because of the small sample size of this study, these findings are preliminary and further comprehensive studies with larger sample sizes are necessary to support the results.

Epigenetic aberrations could be considered as the motivating phenomena in the CRC pathogenesis, and these epigenetic events are accompanied with genetic modifications to elevate the progression of normal colorectal cells to cancer and metastatic cells [14]. It has been found that DNA methylation in collaboration with histone deacetylation results in the epigenetic silencing of tumor-associated genes [12]. As the key enzymes that catalyze these epigenetic processes, HDACs and DNMTs exert crucial roles in the expression regulation of the gene which contributed to carcinogenesis of CRC [41]. Therefore, clarifying the molecular mechanisms through DNA methylation and histone modifications acts as driver events in CRC pathogenesis and could be considered as emerging research approaches to recognize the molecular therapeutic targets for CRC [14]. 

In this study, we described the upregulation of *HDACs* (*HDAC1-4* and *SIRT1*) and three functional *DNMTs* (*DNMT1, DNMT3a* and *DNMT3b*) in 10 sporadic CRC patients using qRT-PCR assays. Among the histone modifying enzymes, histone deacetylases are the most widely characterized proteins with critical roles in the development of CRC [14]. It has been proposed that the transcriptional silencing of tumor suppressor genes via upregulation of *HDACs* could be considered as a usual process in tumor development and progression [42]. Weichert et al. reported that 36.4%, 57.9%, and 72.9% of CRC patients had the *HDAC1*, *HDAC2*, and *HDAC3* over-expressions, respectively. As the transcript levels were significantly increased in poorly differentiated and proliferating cancers, the high levels of *HDAC* expression are correlated with reduced survival of patients. Sirtuin 1 is a class III HDAC whose overexpression was found in 37% of CRC cases and is predominantly correlated with MSI and CIMP-high CRCs. Altogether, these findings propose the histone modification patterns and histone modifying enzymes as biomarkers and chemo-preventive targets in CRC [43]. 

As the best-known epigenetic modifier enzymes [12], overexpression of *DNMTs* has been reported in CRC cases compared with their normal tissues [23, 24, 44, 45]. *DNMT* transcript levels might also be considered as markers since it was found that *DNMT1* was upregulated in 42% of CRC patients [46]. It has been demonstrated that in various types of cancers, *DNMT1* and *DNMT3B* act as the leading catalyzers of TSGs methylation silencing [41]. Moreover, in colon cancer cells, the epigenetic silencing of *CDKN2A *and *MLH1 *genes was also correlated with enhanced levels of *DNMT1* and *DNMT3B* [47]. 

Interestingly, our findings also demonstrated that for some patients, the increase of transcript levels of a number of studied genes was much greater as compared to other patients. As previous studies demonstrated, the expression of *HDACs *and *DNMTs *was significantly related to the tumor grade, stage, and differentiation status [48-50]. This revealed that the overexpression of the *HDACs* and *DNMTs* was related to cancer progression and the enzymes might have been the biomarkers of tumor proliferation and aggressiveness [48]. Based on these results, it could be concluded that the greater transcript levels observed in some cases might result from their higher tumor stage or the lower differentiation status of tumors.

Our results demonstrated that the levels of *DNMTs* mRNA were different in the ten colorectal tumors. In agreement with some previous reports [23, 51], our findings also indicated no correlation between *DNMTs* overexpression and CpG islands hypermethylation in CRC patients. It could be concluded that in CRC patients, the gene-specific promoter hypermethylation is not dependent on the *DNMTs* transcript levels and is regulated through other processes. However, due to the small sample size of the current study, further large scale studies are required to determine the importance of *DNMTs* expression in CpG islands DNA hypermethylation in CRC and other human cancers.

### Acknowledgment:

The authors would like to acknowledge Shiraz University of Medical Sciences for financially supporting this study (Grant No. 27866). The authors would also like to appreciate Dr. Nasrin Shokrpour at the Research Consultation Center (RCC) of Shiraz University of Medical Sciences for her valuable assistance in English editing of this manuscript.

### Conflict of Interest:

The authors have no conflict of interests related to this publication.

### Authors’ Contribution:

MN was involved in investigation, data analysis, and writing- original draft. FN was contributed to methodology, review & editing. SVH was contributed to sample collection and conceptualization. MZ was involved in validation, project administration. PM was involved in methodology, resources, supervision, review & editing. 
